# Editorial: Modulation of behavioral outcomes by conditioning competing states, valences, or responses

**DOI:** 10.3389/fnbeh.2022.959704

**Published:** 2022-08-23

**Authors:** Jacqueline K. Rose, Kristin M. Scaplen, Sheri J. Y. Mizumori, Adam C. Roberts

**Affiliations:** ^1^Behavioral Neuroscience Program, Department of Psychology, Western Washington University, Bellingham, WA, United States; ^2^Department of Psychology, Bryant University, Smithfield, RI, United States; ^3^Department of Neuroscience, Brown University, Providence, RI, United States; ^4^Center for Health and Behavioral Sciences, Bryant University, Smithfield, RI, United States; ^5^Program in Neuroscience, Department of Psychology, University of Washington, Seattle, WA, United States; ^6^Department of Psychology, California State University, Fullerton, CA, United States

**Keywords:** conditioning, behavioral flexibility, counter-conditioning, opposing valence, opposing responses, opposing states, stimulus integration, response flexibility

## Introduction

At any moment, an organism encounters a wide array of stimuli that have the potential to drive learning and behavior. The majority of experience-dependent plasticity (a.k.a., learning) research focuses on investigating the parameters and mechanisms activated by pairing a novel stimulus with an established stimulus-response circuit, the crux of associative conditioning. However, different stimuli can often activate competing and/or opposing neural circuits. There are fewer recent studies that investigate how established circuits that produce opposing responses, states, or valences are integrated in learning. How the activation of opposing circuits are integrated and ultimately influence behavior will significantly inform our understanding of behavioral flexibility, where a response is modulated because of changing internal and external conditions. Furthermore, the associations of competing circuits have the potential for clinical significance as human behavioral states may be subject to the same conditioning and counter-conditioning principles. In this special issue, we feature an assortment of research and reviews that explore the “Modulation of Behavioral Outcomes by Conditioning Competing States, Valences or Responses” at many levels of investigation. This collection expands our understanding of how learning occurs between previously established and opposing circuits by highlighting recent findings and advances.

## Circuit mechanisms

Gil-Lievana et al. and Laurent et al. investigated how learning is influenced by the salience and affective valence of incoming stimuli, respectively. Gil-Lievana et al. implicate VTA dopaminergic input to the insular cortex in consolidating taste recognition memory and identify a specific role for D1-like receptors in consolidating aversive, but not appetitive, taste memory. Laurent et al. report that “transreinforcer blocking” occurs with a positive valence, a phenomenon previously only demonstrated with negative valence, by using a stimulus that indicates the omission of an aversive US when presented in conjunction with a new stimulus and food presentation. Together, these studies show how opposing stimuli can rely on different signaling mechanisms, as is the case of stimulus salience, but produce a similar behavioral learning result, as with affective valence.

Pribic et al. and Orr et al. report that locomotor response pathways are also differentially influenced by either opposing response co-activation or by opposing internal states determined by social status, respectively. Pribic et al. begin to uncover how association of stimuli that drive opposing motor responses modifies locomotor behavior by taking advantage of the well-described neural circuitry of *C. elegans*. Pribic et al. show that pairing stimuli that produce opposite directional movement, results in a novel “pause” (cessation of locomotion) response to a stimulus that previously activated a backward locomotion pathway. Orr et al. found that the opposing states imposed by social hierarchy result in behavioral differences in locomotion and startle sensitivity when comparing dominant and subordinate Zebrafish (*Danio rerio*). Endocannabinoid signaling mediated a switch in locomotion strategy that was pharmacologically reversible, demonstrating that opposing social status can modify locomotion strategy. These studies provide examples of how multiple, competing signals drive response plasticity.

## Behavioral flexibility

Animals have evolved to show adaptive behavioral responses that promote survival and reproduction. Behavioral flexibility allows animals to adjust responses to a changing environment. Hones and Mizumori describe a role for the lateral habenula (LHb) in facilitating response flexibility. LHb is postulated to drive alternative choices, behaviors, and/or internal state conditions by regulating hippocampal theta, a neural state associated with flexible associations, exploration, and greater attention and arousal. Accordingly, behavioral flexibility reflects not only the selection or competition between actions but also changes in neural state that enable consideration of different response options.

Devineni and Scaplen provide an overall conceptual framework for studies of behavioral flexibility in *Drosophila*, including the delineation of general principles of neural circuit organization and modulation that underlie behavioral flexibility across different systems, principles that are likely to extend to other species. Ortu and Bugg describe a model by which different response options or systems might arise and then ultimately, how the response is expressed and the decision for that expression comes about. The latter is proposed to depend on a striatal-thalamo-cortical circuit. Nemchek et al. report that responses are also impacted by predictive stimuli that occur prior to a learning assay by describing the influence that novel vs. familiar pre-test transportation cues can have on novel object exploration.

Overall, these contributions on the neural and behavioral mechanisms of behavioral flexibility inform future therapeutic interventions for disorders of behavioral control that are characterized by response inflexibility, such as, addiction and depression.

## Clinical significance

Understanding the integration of opposing circuits can provide possible avenues for novel therapeutic approaches. Desrochers et al. propose a dual-system approach, which comprises the competing processes of reward processing and inhibitory control, to understand the mechanisms of impulsivity control. They review the role of serotonin signaling in modulating systems that drive and inhibit motivated behaviors respectively and suggest that pathological impulsivity likely arises because of the imbalance between these systems. Steinman et al. report that interventions that draw on the integration of opposing states to maximize the benefits of a learning-based therapy (novelty-facilitated extinction) do not uniformly translate to a sample from a clinical population.

## Conclusions

These reports provide insights into how activation of opposing stimulus-response circuits can modulate future behavioral responses as well as how different qualities of a stimulus, an organism's state, or the environment influences the modulation of response circuitry. What plasticity mechanisms are involved when two or more established response circuits are co-activated remains to be clarified. Furthermore, how a conglomerate of circuits reconciles these competing signals to adapt to changing conditions requires further investigation. As we develop our understanding of these signal integration and potential decision mechanisms, future clinical applications will benefit from a more nuanced understanding of experience-dependent plasticity that incorporates how previous experience is coded within a circuit.

## Author contributions

All authors made a substantial, direct, and intellectual contribution to the work and approved it for publication.

## Funding

KS was supported by the Rhode Island Institutional Development Award (IdeA) Network of Biomedical Research Excellence from the National Institute of General Medical Sciences of the National Institutes of Health under grant number P20GM103430, the Rhode Island Foundation Medical Research Fund 20210957, and the generous support of the Center for Health and Behavioral Sciences at Bryant University.

## Conflict of interest

The authors declare that the research was conducted in the absence of any commercial or financial relationships that could be construed as a potential conflict of interest.

## Publisher's note

All claims expressed in this article are solely those of the authors and do not necessarily represent those of their affiliated organizations, or those of the publisher, the editors and the reviewers. Any product that may be evaluated in this article, or claim that may be made by its manufacturer, is not guaranteed or endorsed by the publisher.

